# Diagnostic Challenges and Treatment Options for Mucocle of the Appendix: A Comprehensive Review

**DOI:** 10.7759/cureus.66142

**Published:** 2024-08-04

**Authors:** Vasundara Gopalan, Imran Ali Khan, Anup A Zade, Geetika Malhotra, Shubham Durge, Yashraj Jain, Sai Goutham Rekavari

**Affiliations:** 1 General Surgery, Jawaharlal Nehru Medical College, Datta Meghe Institute of Higher Education and Research, Wardha, IND

**Keywords:** prognostic factors, treatment strategies, diagnostic challenges, mucinous cystadenocarcinoma, mucinous cystadenoma, appendiceal mucocle

## Abstract

Mucocles of the appendix, encompassing mucinous cystadenomas and mucinous cystadenocarcinomas, represent rare but clinically significant appendiceal lesions characterized by the accumulation of mucin within the appendix lumen. This review explores the diagnostic complexities and treatment strategies associated with mucocles, emphasizing the importance of its accurate recognition and management. Diagnostic challenges arise due to overlapping symptoms with acute appendicitis and other appendiceal pathologies, necessitating a multidimensional approach that includes imaging, histopathological analysis, and clinical correlation. Treatment options range from appendectomy for benign lesions to more extensive surgical procedures, such as right hemicolectomy for malignant forms. Prognostic factors, including histological subtype and tumor size, influence treatment decisions and long-term outcomes. By synthesizing current evidence and clinical insights, this review aims to provide a comprehensive framework for clinicians to navigate the complexities of mucocles of the appendix, offering perspectives that can guide effective management and future research endeavors.

## Introduction and background

A mucocle of the appendix, also referred to as appendiceal mucinous cystadenoma or mucinous cystadenocarcinoma, denotes a rare pathological condition characterized by the accumulation of mucin within the appendix [1]. This condition presents diagnostic challenges due to its diverse clinical presentations and histopathological variations. While mucinous cystadenomas are typically benign, mucinous cystadenocarcinomas can exhibit a malignant potential, necessitating careful diagnostic differentiation and management [2]. The exact incidence of mucocles of the appendix is relatively low, constituting a small fraction of all appendiceal lesions [3]. Mucinous cystadenomas are more commonly encountered than their malignant counterparts, with prevalence rates showing a slight predilection toward females and specific age groups. Understanding the epidemiology of mucocles is crucial for clinicians, as timely recognition can influence treatment decisions and outcomes [4].

Recognizing the diagnostic challenges associated with mucocles is paramount in clinical practice [1]. The symptoms of mucocles often overlap with acute appendicitis and other appendiceal pathologies, complicating its accurate diagnosis. Delayed or incorrect diagnosis may lead to complications such as appendiceal rupture or progression to malignancy in cases of cystadenocarcinomas. Therefore, a nuanced approach to diagnostic imaging, histopathological examination, and clinical correlation are essential for optimal patient care [1]. The primary objective of this review is to provide a comprehensive analysis of mucocles of the appendix, focusing on diagnostic strategies, treatment modalities, and prognostic indicators. By synthesizing current literature and clinical experiences, this review aims to elucidate the complexities surrounding mucocles, offering insights that can inform clinical decision-making and future research directions.

## Review

Anatomy and pathophysiology

Anatomy of the Appendix

The appendix is a narrow, blind-ended tube extending from the posteromedial surface of the cecum, approximately 2-3 cm below the ileocecal valve. Its length varies greatly, ranging from 2 to 20 cm, and the mesoappendix, a fold of mesentery, supports it. The tip of the appendix can be situated in various positions within the abdominal cavity, with the most common location being retrocecal (65-70%) [5]. The appendix contains a substantial amount of lymphoid tissue, which peaks around 20 years of age. It receives its arterial supply from the appendicular artery, a branch of the ileocolic artery, and its venous drainage occurs through the corresponding appendicular vein. Lymphatic drainage is directed to the ileocolic lymph nodes. Innervation is provided by sympathetic and parasympathetic branches of the autonomic nervous system via the ileocolic branch of the superior mesenteric plexus [5].

Pathophysiological Basis of Mucocle Formation

The pathophysiological basis of mucocele formation involves disrupting or obstructing the excretory ducts of salivary glands, leading to the extravasation of mucus into the surrounding soft tissues. The most common cause is trauma, such as a crush-type injury or severance of the excretory duct of a minor salivary gland, often in the lower lip. This results in the leakage of mucus from the duct into the adjacent tissues [6]. Other causes include chronic inflammation and periductal scarring leading to duct obstruction, salivary gland sialolithiasis (stones) obstructing the duct, prior surgery or trauma from oral intubation, and congenital causes like birth trauma affecting the oral cavity. In some cases, mucosal inflammation can lead to blockage, dilation, and rupture of the salivary duct, causing subepithelial fluid spillage, even without clear traumatic etiology [7]. Increased enzymes like matrix metalloproteins and plasminogen activators in mucoceles may enhance the accumulation and invasive nature of the extravasated mucus [8].

Factors Contributing to Mucocle Development

**Figure 1:**
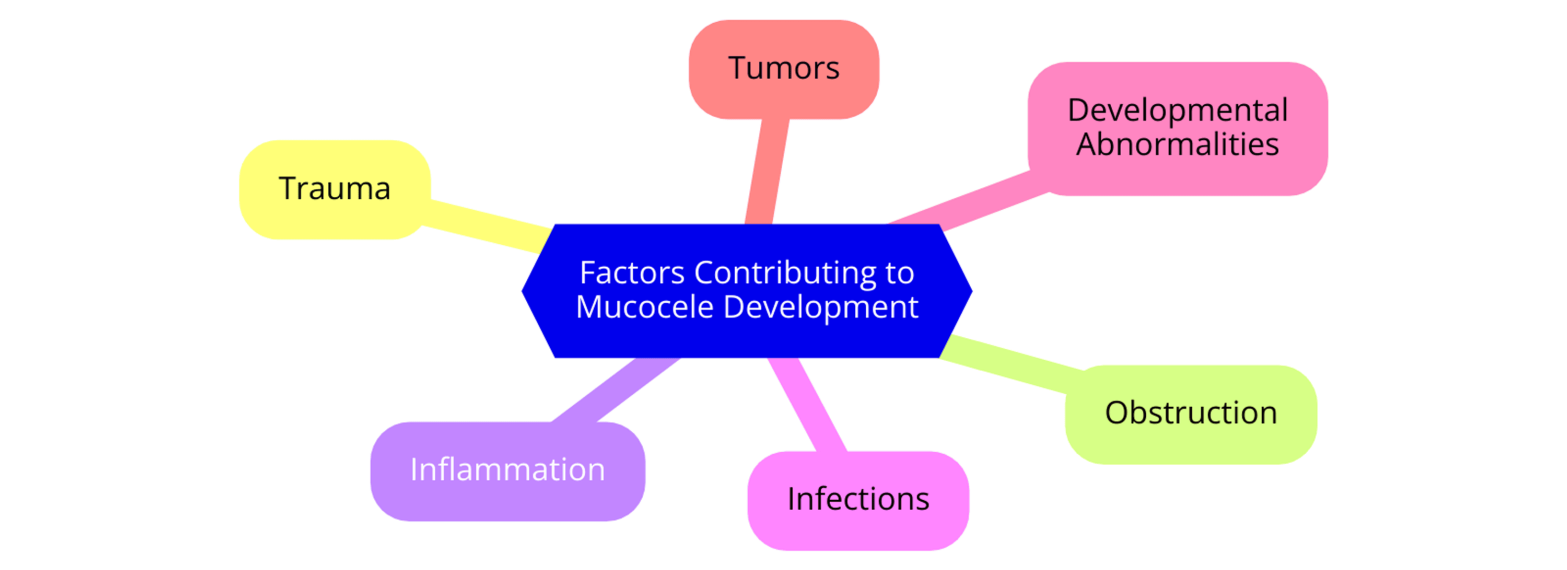
Factors contributing to mucocele development Image Credit: Dr Vasundara Gopalan

The development of an appendiceal mucocele is primarily driven by obstruction and the abnormal accumulation of mucinous substance within the appendiceal lumen. This obstructive dilation is the defining characteristic of a mucocele [9]. The underlying cause of the obstruction can range from a simple retention cyst to a malignant neoplasm. Benign retention cysts typically do not exceed 2 cm in diameter, while malignant mucoceles are often larger. Factors such as mural nodularity, irregular wall thickening, and the overall diameter of the lesion may suggest a malignant etiology. Still, these features are not definitive in preoperatively distinguishing between benign and malignant causes [10]. Prompt diagnosis of a mucocele is crucial, as a ruptured neoplastic mucocele can lead to the development of a serious complication known as pseudomyxoma peritonei. This condition, characterized by disseminating mucin-producing cells throughout the peritoneal cavity, dramatically worsens the patient's prognosis. Therefore, early recognition of the mucocele and appropriate surgical management are essential to prevent this devastating outcome [11]. Factors contributing to mucocele development are illustrated in Figure 1.

Clinical presentation

Symptoms and Signs

The clinical presentation of an appendiceal mucocele can be quite variable and nonspecific. The most common symptom is pain in the right lower quadrant (RLQ), which can be acute or chronic and may mimic appendicitis. Patients may also experience a palpable abdominal mass in the RLQ, nausea, vomiting, weight loss, changes in bowel habits, anemia, and fever. On physical examination, there may be tenderness to palpation in the RLQ, generalized abdominal tenderness, and a palpable lump or mass in the RLQ [1]. However, the clinical presentation is often delayed and atypical, making preoperative diagnosis challenging. Many mucoceles are discovered incidentally during imaging or surgical procedures for other conditions. Symptoms can be vague, such as lower abdominal pain or a palpable lump, which can mimic other conditions like ovarian cysts. Failure to make an early preoperative diagnosis increases the risk of complications like rupture and the development of pseudomyxoma peritonei, which significantly worsens the prognosis [12]. The symptoms and signs associated with appendiceal mucocele are varied and often nonspecific. A high index of suspicion is necessary, especially in cases with atypical presentations or incidental findings, to ensure a prompt diagnosis and appropriate management. Early recognition of this condition is crucial to prevent serious complications and optimize patient outcomes [13].

Differential Diagnosis with Other Appendiceal Lesions

Distinguishing between mucocele and acute appendicitis can be particularly challenging, as both conditions can present with similar symptoms of RLQ pain. However, mucoceles are often larger in size compared to acute appendicitis and may show mural calcifications on imaging, which can help differentiate the two conditions [14]. Differentiating between benign and malignant causes of mucocele preoperatively is another significant challenge. Factors such as mural nodularity, irregular wall thickening, and lesion diameter are not always reliable in making this distinction. Generally, benign retention cysts typically do not exceed 2 cm in diameter, while malignant mucoceles are often larger. However, there can be significant overlap, and a definitive diagnosis may only be possible after surgical resection and histopathological analysis [15]. Mucoceles can also resemble other cystic lesions in the right lower abdomen, such as ovarian cysts, further complicating the diagnostic process. On CT imaging, a well-circumscribed, low-attenuation, spherical, or tubular mass contiguous with the cecum base is suggestive of a mucocele. However, this radiological appearance is not pathognomonic, and a high index of suspicion is required to make the correct diagnosis [16].

Diagnostic modalities

Imaging Techniques (Ultrasound, CT Scan, MRI)

Ultrasound (US) is often the first-line imaging tool for appendiceal lesions. However, ultrasound may not always identify the origin of the tumor, necessitating additional imaging modalities. CT scans can show mucoceles as well-circumscribed, low-attenuation, spherical, or tubular masses contiguous with the base of the cecum. The presence of curvilinear mural calcification suggests the diagnosis, although it is seen in less than 50% of cases. CT can help differentiate mucoceles from other cystic lesions in the right lower quadrant, but distinguishing between benign and malignant causes preoperatively can be difficult [17]. Magnetic resonance imaging (MRI) can provide detailed imaging of the appendix and surrounding tissues. It is particularly useful for differentiating between benign and malignant mucoceles, as it can show mural nodularity and irregular wall thickening, which are associated with malignancy. While ultrasound is often the initial imaging modality, CT and MRI are complementary techniques that provide important information for diagnosing mucoceles and determining the appropriate surgical management. However, a definitive diagnosis is often only made after surgical resection and histopathological examination [3].

Laboratory Findings (Tumor Markers, Inflammatory Markers)

Mucocele of the appendix is a rare condition characterized by the obstructive dilation of the appendix due to the abnormal accumulation of mucinous substance within its lumen. While diagnosing a mucocele can be challenging, certain laboratory findings may provide useful information [2]. Tumor markers can help evaluate the underlying etiology of a mucocele. Mucoceles with a neoplastic origin, such as mucinous neoplasms, may demonstrate elevated levels of carcinoembryonic antigen (CEA), CA 19-9, and CA-125. Elevated CEA levels have been reported in some cases of mucinous neoplasms of the appendix. Measuring and monitoring these tumor marker levels can offer insights into the stage and progression of the tumor, although they are not definitive diagnostic tools [18]. In contrast, inflammatory markers are typically not specific for mucoceles. Laboratory values are usually nonspecific and may not aid in the preoperative diagnosis of a mucocele. The definitive diagnosis of a mucocele ultimately relies on the histopathological examination of the surgical specimen, as the imaging features can sometimes mimic other cystic lesions in the right lower abdominal quadrant [6]. While tumor markers like CEA, CA 19-9, and CA-125 can be elevated in neoplastic mucoceles, the laboratory findings are generally nonspecific. These tumor markers can provide useful information about the underlying etiology and disease progression, but they should be interpreted in conjunction with other clinical and radiological findings. The definitive diagnosis of a mucocele remains dependent on the histopathological analysis of the surgical specimen [18].

Role of Diagnostic Laparoscopy

The role of diagnostic laparoscopy in managing mucoceles of the appendix is multifaceted. Laparoscopy enables the direct visualization of the abdominal and pelvic organs, facilitating diagnosis in cases where symptoms or imaging findings are ambiguous [19]. Specifically for mucoceles, laparoscopy proves valuable when traditional imaging like ultrasound or CT scans fail to definitively distinguish between benign and malignant causes. Direct inspection allows confirmation of the mucocele's presence and provides critical details such as size, wall thickness, and any nodularity or irregularities [20]. Moreover, diagnostic laparoscopy plays a pivotal role in staging and assessing operability, especially for suspected malignant mucoceles. By visualizing disease extent and involvement of adjacent structures, surgeons can determine the optimal surgical approach - ranging from simple appendectomy to more extensive procedures like right hemicolectomy - as warranted [21]. Occasionally, diagnostic laparoscopy seamlessly transitions into therapeutic intervention, enabling immediate treatment of the identified mucocele. This approach minimizes the need for additional surgeries, reducing patient burden [22].

Histopathological classification

Histopathological Features and Implications for Prognosis

Mucoceles of the appendix are histologically classified into four main types, each with distinct features and implications for prognosis. The first type is the simple retention cyst, caused by appendiceal lumen obstruction leading to mucus accumulation. These cysts are typically small (<2 cm), lined by flattened or attenuated epithelium without atypia, and have an excellent prognosis with appendectomy [1]. The second type is mucosal hyperplasia, characterized by hyperplasia of the appendiceal mucosa. These lesions may display thickened folds and papillary projections, but the epithelium is usually non-dysplastic. The prognosis with appendectomy for mucosal hyperplasia is generally favorable [23]. The third type is mucinous cystadenoma (adenoma), a neoplastic proliferation of appendiceal mucosa. These lesions are larger (>2 cm), lined by dysplastic epithelium with varying degrees of atypia. Considered low-grade malignancies, mucinous cystadenomas typically have a five-year survival rate of 91%-100% with appendectomy [24]. The fourth and most serious type is mucinous cystadenocarcinoma (adenocarcinoma), a malignant neoplasm with infiltrative growth into the appendiceal wall. These lesions exhibit high-grade cytologic atypia, increased mitotic activity, and are associated with pseudomyxoma peritonei. The prognosis for mucinous cystadenocarcinoma is poor, with a five-year survival rate of only 25% [25]. Distinguishing between these subtypes is critical for guiding surgical management. Simple retention cysts and mucosal hyperplasia typically warrant appendectomy alone. In contrast, neoplastic mucoceles (cystadenomas and cystadenocarcinomas) may necessitate more extensive resection, such as right hemicolectomy, to minimize recurrence and metastasis risk. Rupture of a neoplastic mucocele can lead to pseudomyxoma peritonei, significantly worsening prognosis. Therefore, prompt diagnosis and appropriate surgical intervention are crucial for managing appendix mucoceles [26].

Differential diagnosis

Distinguishing Mucocle From Other Appendiceal Lesions

Appendiceal mucoceles are a rare condition characterized by the obstructive dilation of the vermiform appendix due to abnormal accumulation of mucinous substance in its lumen. While various imaging tests such as CT, ultrasound, colonoscopy, and MRI can demonstrate characteristic features such as a cystic structure with a tubular shape, blind-ending, and contiguous with the cecum, these typical findings are only observed in about 40% of the cases before surgery. Diagnosis remains challenging because mucoceles can mimic several other conditions including acute appendicitis, malignant neoplasms, ovarian cysts, mesenteric cysts, lymphoceles, and inflammatory diseases. In approximately 60% of the cases, the diagnosis is made intraoperatively or incidentally, confirmed by histopathological analysis [1]. Key imaging features that suggest malignancy include mural nodularity and irregular wall thickening. Benign retention cysts typically measure less than 2 cm in short-axis diameter. However, variables such as lesion diameter, internal content attenuation, septations, and wall calcifications do not reliably distinguish between benign and malignant mucoceles. Treatment approaches vary based on the underlying cause. Simple cystadenomas are usually managed with appendectomy, while neoplastic mucoceles require more aggressive resection, often involving right hemicolectomy. Ruptured mucoceles can lead to pseudomyxoma peritonei, significantly impacting prognosis, underscoring the importance of timely diagnosis and treatment [3]. On CT scans, appendiceal mucoceles typically appear as well-defined, low-attenuation masses that are spherical or tubular, contiguous with the base of the cecum. Curvilinear mural calcifications, although present in less than 50% of the cases, can suggest a mucocele. Ultrasound findings are nonspecific but may reveal a porcelain appendix or demonstrate an "onion skin sign" indicating concentric layers of lamellated mucin. While mucoceles can mimic acute appendicitis clinically, key imaging features such as a larger appendiceal diameter, lower white blood cell count, and microscopic hematuria can aid in the preoperative differentiation. A comprehensive assessment combining clinical, laboratory, and radiological findings is crucial for accurate diagnosis [27].

Treatment strategies

Surgical Management Options (Appendectomy, Right Hemicolectomy)

Mucocele of the appendix is a rare condition characterized by the obstructive dilation of the vermiform appendix due to abnormal accumulation of mucinous substance in its lumen. Treatment approaches vary depending on the underlying cause. Simple cystadenomas, which are benign, are typically managed with appendectomy. This surgical procedure is also performed incidentally during operations for other concurrent conditions [28]. In contrast, neoplastic mucoceles, such as cystadenocarcinomas and other malignant forms, require more aggressive management. The treatment of choice for these cases is right hemicolectomy. This procedure may involve additional interventions like debulking for pseudomyxoma peritonei, total abdominal hysterectomy with salpingo-oophorectomy, segmental colonic resections, and cholecystectomy when necessary [29]. While laparoscopic appendectomy and right hemicolectomy are feasible for selected cases, the laparoscopic approach is generally avoided due to the risk of rupture and peritoneal spillage, which can lead to pseudomyxoma peritonei. The decision between appendectomy and right hemicolectomy depends on the suspicion of malignancy and the extent of the disease. Appendectomy is appropriate for benign lesions, whereas right hemicolectomy is necessary for malignant mucoceles to ensure their complete removal and reduce the risk of recurrence [30].

Decision-Making Algorithms Based on Histopathological Findings

Histopathological image analysis plays a critical role in achieving accurate diagnoses and prognoses across various diseases. Increasingly, machine learning techniques are being integrated to aid pathologists in this endeavor. The typical workflow involves preprocessing whole slide images, applying diverse machine learning algorithms, and categorizing tissue morphology using multicriteria decision-making techniques [31]. Unaided human-based classification relies on pathologists using decision tree-based approaches, leveraging their visual examination and diagnostic expertise to make reproducible decisions [32]. In contrast, machine learning employs advanced algorithms like deep learning to automate and enhance the accuracy of histopathological diagnosis. These methods can identify diagnostically relevant regions of interest within large-scale digital images, enabling a comprehensive microscopic pathology image feature analysis and prognosis prediction [32]. However, digital pathology poses unique challenges that require attention. Collaborative analysis of multi-gigapixel imaging data using tools such as Cytomine is crucial for managing the scale and complexity of digital pathology tasks [33]. Additionally, tracking pathologists' interactions with digital whole slide images - such as mouse cursor movements and eye tracking data - offers valuable insights into decision-making processes, aiding in algorithm refinement and validation [33]. Ultimately, decision-making algorithms in histopathological image analysis synergize human expertise with machine learning capabilities. While pathologists contribute their interpretative skills, machine learning methods automate processes and enhance diagnostic accuracy. Addressing the specific challenges of digital pathology ensures these algorithms reliably support clinical decision-making and patient care [34].

Role of Laparoscopic Versus Open Surgery

The debate over laparoscopic versus open surgery for treating appendiceal mucoceles revolves around distinct advantages and disadvantages associated with each approach, influenced by factors such as patient condition, surgeon experience, and mucocele characteristics [35]. Laparoscopic surgery offers several benefits. It is minimally invasive, resulting in smaller wounds, shorter hospital stays, and reduced postoperative pain and complications. Patients generally experience faster recovery and improved cosmetic outcomes due to smaller incisions. Studies have demonstrated comparable oncologic outcomes with laparoscopic resections, including excellent survival rates for benign mucoceles [35]. However, laparoscopic surgery requires specialized training and advanced equipment, which may not be universally available. There is also a risk of mucin spillage during resection, particularly concerning for neoplastic mucoceles as it can lead to pseudomyxoma peritonei. In some cases, laparoscopic procedures may need conversion to open surgery due to challenges like inadequate resection margins or unexpected complications [36]. Open surgery, in contrast, provides direct visualization of the surgical field, which can be advantageous for complex anatomical scenarios or cases involving severe inflammation, scarring, or bleeding. Most surgeons are proficient in open techniques, which have been traditionally used for decades [37]. However, open surgery is more invasive, involving a larger incision that leads to more tissue trauma, longer hospital stays, and slower recovery times. The larger incision also results in more visible scarring, which can be a consideration for some patients [37].

Prognostic factors

Factors Influencing Prognosis

The prognosis of appendiceal mucoceles varies significantly based on their histological subtype. Benign mucoceles, such as simple retention cysts, generally have an excellent prognosis, with a five-year survival rate of 91%-100% following standard appendectomy. These lesions are typically smaller than 2 cm in diameter and lack concerning features like mural nodularity or irregular wall thickening [12]. In contrast, malignant mucoceles present greater challenges. Mucinous cystadenomas may recur but often can be effectively managed with appendectomy alone. However, mucinous cystadenocarcinomas carry a more serious prognosis, with an increased risk of recurrence and spread, especially if there is evidence of extra-appendiceal acellular mucin containing neoplastic epithelium. These malignant lesions tend to be larger than benign ones [11]. One of the most severe complications associated with mucoceles is the development of pseudomyxoma peritonei, which can occur if a neoplastic mucocele ruptures. This condition significantly worsens the prognosis, with five-year survival rates ranging from 53% to 75%. Prompt diagnosis and appropriate treatment, which may involve more extensive procedures like right hemicolectomy, are critical in such cases to prevent this devastating outcome [11].

Long-term Outcomes and Recurrence Rates

The long-term outcomes and recurrence rates for appendiceal mucoceles vary significantly depending on the underlying etiology and treatment approach. Benign mucoceles, such as simple retention cysts, have an excellent prognosis with a five-year survival rate of 91%-100% following standard appendectomy. These lesions typically do not recur after surgical removal, making appendectomy curative in most cases [12]. In contrast, malignant mucoceles, including mucinous cystadenomas and mucinous cystadenocarcinomas, pose a higher risk of recurrence and dissemination. Patients with neoplastic epithelium containing extra-appendiceal acellular mucin are particularly vulnerable, with recurrence or progression occurring in 33%-75% of cases. The five-year survival rate for mucinous cystadenocarcinomas is notably lower at 25%, reflecting the aggressive nature of these malignancies [25]. Pseudomyxoma peritonei, a serious complication that can arise from a ruptured mucocele, significantly worsens the prognosis, with a five-year survival rate ranging from 53% to 75%. Treatment strategies vary depending on the underlying pathology. Nonneoplastic mucoceles are typically managed with appendectomy, which is generally curative. In cases of malignant mucoceles, right hemicolectomy is often recommended to ensure complete removal and reduce the risk of recurrence. For patients with disseminated peritoneal disease, treatments such as hyperthermic intraperitoneal chemotherapy (HIPEC) following cytoreductive surgery may be employed [38]. Understanding the long-term outcomes and recurrence rates associated with appendiceal mucoceles is essential for guiding treatment decisions and predicting the likelihood of disease progression or recurrence [39].

Case studies and clinical scenarios

Presentation of Clinical Cases Illustrating Diagnostic and Management Challenges

Mucocele of the appendix presents substantial diagnostic challenges, often lacking characteristic imaging findings preoperatively. The first case study illustrates this complexity: a patient with a right iliac fossa mass initially thought to be gynecological. Despite extensive preoperative investigations, the diagnosis of appendiceal mucocele was only established during surgery. The authors emphasize the importance of considering an appendiceal origin in the differential diagnosis of women presenting with a right iliac fossa mass [1]. Similarly, the second case study underscores the difficulty in preoperative diagnosis. A 70-year-old female presented with right iliac fossa pain and nonspecific symptoms. Imaging with ultrasound and CT scan suggested appendiceal mucocele, but intraoperatively, the identification of a broad-based mucocele communicating with the cecum posed challenges that were not evident on imaging. This highlights the significant impact of intraoperative findings on surgical management [40]. The management challenges are further illustrated in the second case study, where intraoperative discovery of a cystic appendiceal mass with a broad base led to a decision for extended right hemicolectomy due to suspected malignancy and the absence of frozen section analysis. Subsequent histopathology confirmed a mucinous cystadenoma with mucocele, demonstrating how intraoperative findings can alter surgical plans [41]. These cases underscore the diagnostic difficulty of appendiceal mucoceles, often misidentified as other pelvic or abdominal masses preoperatively. Intraoperative findings, such as the presence of a wide base communicating with the cecum, are critical in guiding surgical decisions. A high index of suspicion is crucial given the spectrum from benign to malignant mucoceles, necessitating tailored surgical approaches based on intraoperative findings [1].

Lessons Learned From Case Studies

Mucocele of the appendix presents significant diagnostic challenges, often leading to a definitive diagnosis only during surgery or after histopathological examination, as the characteristic features are observed in only about 40% of the cases preoperatively. This diagnostic uncertainty can result in various radiological differentials, including acute appendicitis, malignant tumors, ovarian cysts, mesenteric cysts, lymphoceles, and inflammatory conditions. Imaging modalities such as CT, ultrasound, colonoscopy, and MRI can be helpful, but they frequently fail to conclusively identify the underlying cause [12]. In terms of surgical management, the approach depends on the nature of the mucocele. For benign conditions like simple cystadenomas, appendectomy is typically curative and associated with favorable outcomes. However, for cases involving neoplastic growth or suspicion of malignancy, more aggressive resection such as right hemicolectomy is recommended. Laparoscopic appendectomy is feasible when the mucocele is localized within the appendix and there are no signs of rupture or malignancy. In instances where malignancy or rupture is suspected, open surgical exploration is preferred to minimize the risk of mucus and tumor cell dissemination [42]. Case studies emphasize the critical importance of early diagnosis. Timely recognition is crucial because a ruptured neoplastic mucocele can lead to pseudomyxoma peritonei, a condition that significantly worsens prognosis. Early diagnosis followed by appropriate surgical intervention can mitigate serious complications and improve patient outcomes significantly [43].

Future directions and research implications

Emerging Diagnostic Techniques

Advances in imaging technologies such as PET, MRI, and ultrasound are revolutionizing cancer detection by enabling earlier diagnosis and more accurate staging. These modalities, along with techniques like radiogenomics that integrate imaging data with genomic information, enhance the precision of diagnosis and treatment planning. Biomarkers, including genetic, proteomic, and metabolomic markers, play an expanding role in cancer screening, early detection, and monitoring treatment response. Biosensors capable of detecting these biomarkers are becoming increasingly sophisticated, offering the potential for real-time, minimally invasive cancer diagnosis [44]. The analysis of circulating tumor cells, exosomes, and cell-free tumor DNA in blood samples represents a promising avenue for cancer diagnosis and monitoring, eliminating the need for traditional tissue biopsies. Next-generation sequencing technologies facilitate a comprehensive analysis of these liquid biopsy samples, providing insights into tumor biology and treatment response. Additionally, artificial intelligence (AI) and machine learning (ML) algorithms are being deployed to analyze vast datasets encompassing imaging, genomic, and clinical data. These computational tools are instrumental in developing diagnostic algorithms and decision support systems that enhance personalized and precise cancer care, improving detection accuracy, classification, and prognostication [45].

Advancements in Surgical Approaches and Outcomes

Conventional surgical excision is currently the most widely used and effective treatment for mucoceles. This approach involves a complete removal of the mucocele and its associated salivary gland to prevent recurrence. Surgical excision is well-established, cost-effective, and does not necessitate specialized equipment. However, it demands precise surgical skills to minimize risks such as mucocele rupture during the procedure [46]. Emerging minimally invasive techniques like cryosurgery, laser ablation, and micromarsupialization are gaining attention as alternatives to conventional surgery. These methods aim to provide less invasive options with reduced postoperative complications and quicker recovery times. Cryosurgery, which employs cryogenic agents like liquid nitrogen, can effectively treat mucoceles with minimal swelling and irritation, although healing may take longer. Laser-assisted techniques using argon or Nd:YAG lasers have demonstrated good outcomes with low recurrence rates and high patient satisfaction. Micromarsupialization, involving the placement of a suture through the mucocele, is less invasive but may lead to higher recurrence rates compared to excision [47]. While conventional surgical excision remains the gold standard due to its reliable long-term outcomes and low recurrence rates when performed correctly, minimally invasive therapies offer potential benefits such as reduced invasiveness and faster recovery. However, the comparative long-term efficacy of these newer techniques requires further investigation [48]. A multidisciplinary approach that integrates imaging, surgical expertise, and pathological evaluation is essential for accurate diagnosis and optimal management of mucoceles. In summary, while conventional surgical excision is the established treatment of choice, emerging minimally invasive techniques present promising alternatives that may provide advantages in terms of recovery and patient comfort, though ongoing research is necessary to fully understand their outcomes over time.

## Conclusions

In conclusion, mucocles of the appendix represent a rare but significant clinical entity characterized by the accumulation of mucin within the appendix. The diagnostic journey for these lesions involves navigating through overlapping symptoms with acute appendicitis and other appendiceal pathologies, highlighting the importance of thorough clinical evaluation and appropriate diagnostic modalities. Treatment strategies vary based on histopathological findings, ranging from conservative management for benign mucinous cystadenomas to more aggressive surgical approaches for mucinous cystadenocarcinomas. Prognostic factors such as histological subtype and tumor size influence long-term outcomes, underscoring the need for tailored management approaches. Advances in imaging techniques and molecular diagnostics hold promise for improving early detection and personalized treatment strategies in the future. Continued research efforts are crucial to further refine our understanding of mucocles of the appendix and enhance clinical outcomes for affected patients.
